# Supplementary material for: Enhanced spin–orbit coupling in core/shell nanowires
Source: Nat Commun. 2016 Aug 5;7:12413. doi: 10.1038/ncomms12413 (PMC4980452; doi:10.1038/ncomms12413)
Supplement: Supplementary Information — Supplementary Figures 1-2, Supplementary Notes 1-2 and Supplementary References [file ncomms12413-s1.pdf]

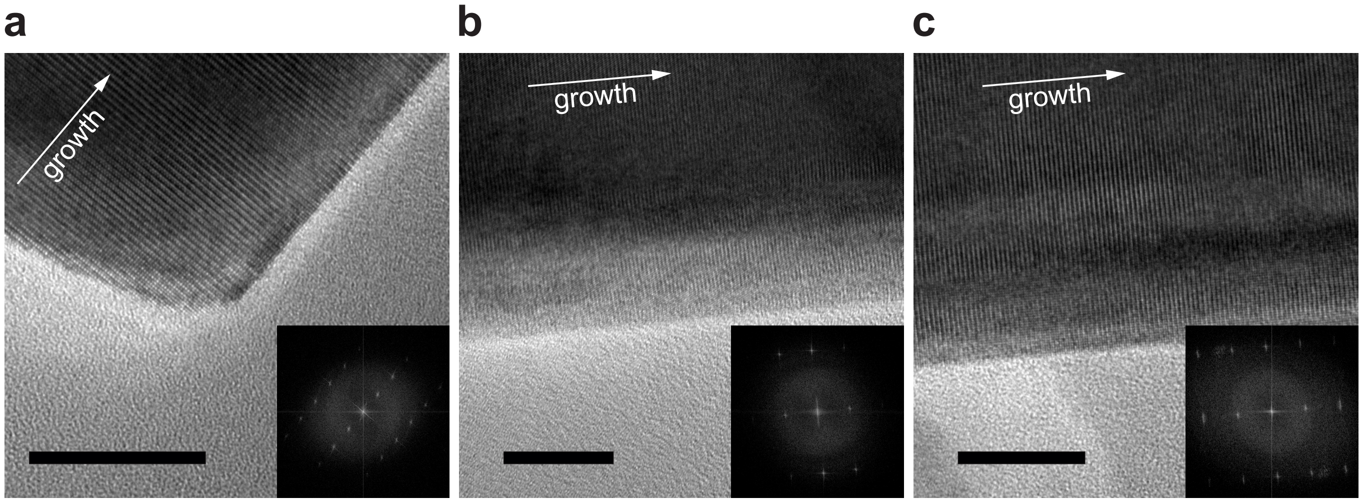

Supplementary Figure 1 | Structural characterization of the NWs. High-resolution transmission electron microscopy (HRTEM) images of a representative GaAs NW, which was comprehensively analyzed with HRTEM over its entire length. The micrographs shown here are exemplarily taken at (**a**) the bottom, (**b**) the center, and (**c**) close to the tip of the NW, each revealing a pure, defect-free WZ GaAs crystal phase—characteristic of the investigated NW sample. The presence of a pure WZ structure is also confirmed by the corresponding patterns in reciprocal space obtained by the fast Fourier transforms of the HRTEM images, which are displayed at the bottom right, respectively. Scale bars, 20 nm.

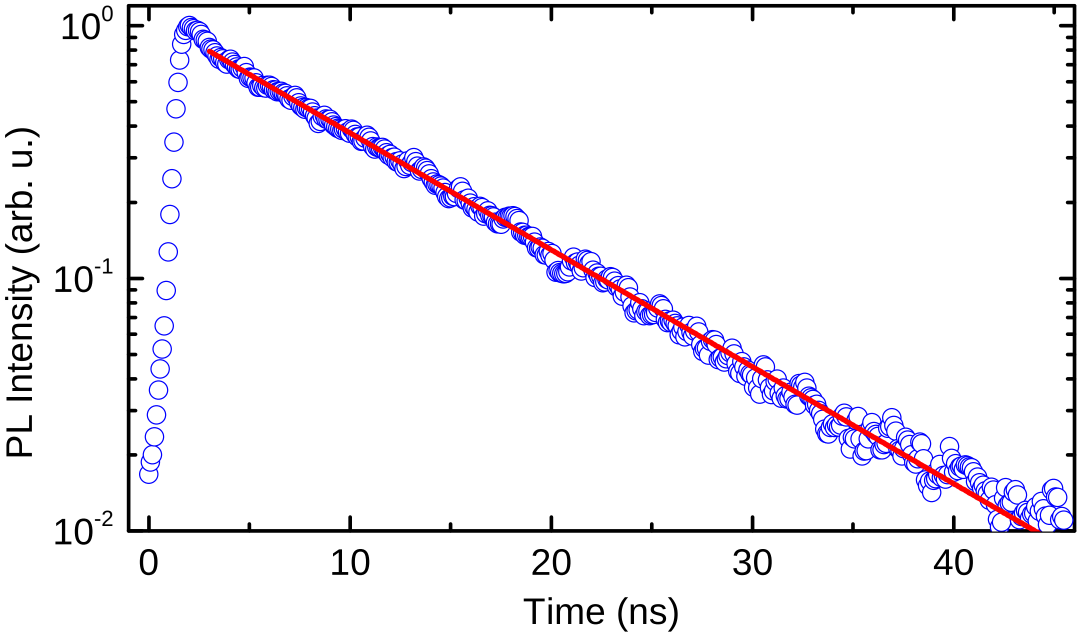

Supplementary Figure 2 | Lifetime measurement of a WZ GaAs NW. Typical time decay of the µ-PL emission from a single, free-standing WZ GaAs NW at 4.2 K. The transient is fitted with a single exponential (continuous red line) consistent with a pure excitonic emission. The extracted free exciton recombination lifetime is 9.4 ns.

Supplementary Note 1 | Spin splitting in bulk wurtzite crystals

The general conduction band spin splitting in binary bulk wurtzite (WZ) semiconductors arises from two contributions, one being linear in the electron's wave vector k while the other one is proportional to the cube k^3. The cubic k^3-dependent term (called Dresselhaus effect) also describes the spin splitting in semiconductors with zincblende structure and is a consequence of the bulk inversion asymmetry (BIA) of the crystal lattice. The k-linear term (called Rashba effect) occurs in the WZ structure due to the hexagonal $\hat{c}$-axis and reflects an intrinsic wurtzite structure inversion asymmetry (WSIA). The relevant SOC Hamiltonian $H_{\mathrm{SO}}^{\mathrm{bulk}}$ with both BIA and WSIA contribution reads as follows[1–11]:

$$H_{\mathrm{SO}}^{\mathrm{bulk}} = H_{\mathrm{SO}}^{\mathrm{BIA}} + H_{\mathrm{SO}}^{\mathrm{WSIA}} = \mathbf{\Omega}_{\mathbf{k}}^{\mathrm{bulk}} \cdot \boldsymbol{\sigma}\,, \tag{1}$$

with

$$\mathbf{\Omega}_{\mathbf{k}}^{\mathrm{bulk}} = \left[\gamma_{\mathrm{e}}\left(bk_z^2 - k_\perp^2\right) + \alpha_{\mathrm{e}}\right] \begin{pmatrix} k_y \\ -k_x \\ 0 \end{pmatrix} = \beta \begin{pmatrix} k_y \\ -k_x \\ 0 \end{pmatrix}\,, \tag{2}$$

where $z\|[0001]$ ($\hat{c}$-axis), $x\|[11\bar{2}0]$, $y\|[1\bar{1}00]$ (cf. Fig. 1c of the manuscript), $k_\perp^2 = k_x^2 + k_y^2$, and $\boldsymbol{\sigma}$ is the vector of the Pauli spin matrices σ_i, $i = x, y, z$. The Dresselhaus coefficient γ_{e}, together with the material parameter b, determines the cubic BIA contribution, while the Rashba coefficient α_{e} gives the strength of the k-linear WSIA contribution. The coefficient $\beta = \left[\gamma_{\mathrm{e}}\left(bk_z^2 - k_\perp^2\right) + \alpha_{\mathrm{e}}\right]$ is then an effective SOC parameter describing the total magnitude of the SO field in bulk WZ.

Supplementary Note 2 | Evaluation of interface-induced SOC for all nanowire sidewall facets

The investigated WZ GaAs nanowires (NWs) exhibit a hexagonal cross section with six equivalent GaAs/AlGaAs core/shell interfaces ($n = 0, 1, 2, \ldots, 5$), which, according to transmission electron microscopy, are oriented along the $\langle 11\bar{2}0\rangle$-directions of the WZ unit cell (cf. Fig. 1 of the manuscript). As outlined in the Discussion of the manuscript, the interface-induced SO field, $\mathbf{\Omega}_{\mathbf{k}}^{\mathrm{int},n}$, resulting from the particular heterointerface n, has the form

$$\mathbf{\Omega}_{\mathbf{k}}^{\mathrm{int},n} = \begin{pmatrix} \alpha_\perp k_z \sin(n\pi/3) \\ -\alpha_\perp k_z \cos(n\pi/3) \\ \alpha_\| \left[k_y \cos(n\pi/3) - k_x \sin(n\pi/3)\right] \end{pmatrix}\,, \tag{3}$$

where the effective SOC parameters $\alpha_\|$ and $\alpha_\perp$ determine the strength of the interfacial contribution parallel and perpendicular to the WZ $\hat{c}$-axis, respectively. Here we note again that, due to the low symmetry $\{11\bar{2}0\}$ NW sidewall facets of the C_{s} point group, $\alpha_\|$ and $\alpha_\perp$ are linearly independent[12,13], while the size of the bulk contribution is given by the single parameter β.

In the absence of external magnetic fields, the dynamics of an electron spin density $\mathbf{S}(t)$, provided that the spin lifetime is longer than the carrier thermalization time, is given by the equation of motion $\dot{S}_i(t) = -\sum_j \Gamma_{ij} S_j(t)$, where Γ_{ij} are components of the spin relaxation rate tensor $\mathbf{\Gamma}$, and $S_{i,j}$ are the average spin components in direction $i, j = x, y, z$. The form of the tensor $\mathbf{\Gamma}$ depends on the spin relaxation mechanism and the symmetry of the system. In a simplified form of the DP spin relaxation theory, Γ_{ij} are determined by the effective magnetic field $\mathbf{\Omega}_{\mathbf{k}}$ via (cf. equation (2) of the manuscript)[14–18]

$$\Gamma_{ij} \sim \left(\delta_{ij}\langle\mathbf{\Omega}_{\mathbf{k}}^2\rangle - \langle\Omega_{\mathbf{k},i}\Omega_{\mathbf{k},j}\rangle\right)\tau_{\mathrm{p}}^*\,, \tag{4}$$

where τ_{p}^* is the momentum relaxation time for an individual electron, δ_{ij} is the Kronecker delta symbol, the angle brackets denote averaging over the momentum distribution of electrons, and

$$\langle\mathbf{\Omega}_{\mathbf{k}}^2\rangle = \sum_i \langle\Omega_{\mathbf{k},i}^2\rangle\,. \tag{5}$$

Taking into account that the electron phase coherence length is smaller than the NW diameter, the respective mean square effective magnetic fields with both bulk and interface contributions add to

$$\langle\Omega_{\mathbf{k},i}\Omega_{\mathbf{k},j}\rangle = \langle\Omega_{\mathbf{k},i}^{\mathrm{bulk}}\Omega_{\mathbf{k},j}^{\mathrm{bulk}}\rangle + \frac{1}{6}\sum_{n=0}^{5}\langle\Omega_{\mathbf{k},i}^{\mathrm{int},n}\Omega_{\mathbf{k},j}^{\mathrm{int},n}\rangle\,. \tag{6}$$

Evaluating Supplementary Equation (4) using Supplementary Equations (2), (3), (5) and (6) by averaging over an isotropic angular distribution of $\mathbf{k}$ shows that $\mathbf{\Gamma}$ is a diagonal tensor with nonzero components Γ_{xx}, Γ_{yy}, and Γ_{zz}, where

$$\Gamma_{xx} = \Gamma_{yy} = \left(\frac{1}{2} + \frac{\alpha_{\parallel}^2}{2\beta^2 + \alpha_{\perp}^2} \right) \Gamma_{zz} \, . \tag{7}$$

DP spin relaxation in WZ GaAs NWs is therefore anisotropic with respect to the NW axis, which corresponds to the WZ $\hat{c}$-axis, while spin relaxation in the plane perpendicular to the NW axis is isotropic.

In the TRPL experiments, the spin component S_z along the $\hat{c}$-axis is detected. In the absence of an external magnetic field ($B_{\text{ext}} = 0$), the exponential decay of S_z corresponds to the zero-field spin lifetime $\tau_{\text{s}}^0 = 1/\Gamma_{zz}$. Applying $\mathbf{B}_{\text{ext}}$ now leads to Larmor precession of the electron spins and an advanced spin dynamics with

$$S_z(t) \propto \mathrm{e}^{-(\Gamma_{yy}+\Gamma_{zz})t/2} \cos\left(\omega_{\text{L}} t\right) \, , \tag{8}$$

where $\mathbf{B}_{\text{ext}} \parallel x$. The measured spin lifetime τ_{s}^B reflects in this case the spin polarization decay with the average decay rate $\Gamma_{\text{eff}} = \left(\Gamma_{yy} + \Gamma_{zz}\right)/2 = 1/\tau_{\text{s}}^B$. Because of Supplementary Equation (7) an averaged relaxation rate $\Gamma_{\text{eff}} = \left(\frac{3}{4} + \frac{\alpha_{\parallel}^2}{4\beta^2 + 2\alpha_{\perp}^2} \right) \Gamma_{zz}$ is obtained due to anisotropic DP spin relaxation. Hence the relative relationship between the strengths of the SOC contributions parallel ($\alpha_{\parallel}$) and perpendicular (β and $\alpha_{\perp}$) to the NW axis can be described by

$$\frac{\alpha_{\parallel}^2}{2\beta^2 + \alpha_{\perp}^2} = 2\frac{\tau_{\text{s}}^0}{\tau_{\text{s}}^B} - \frac{3}{2} \, . \tag{9}$$

Accordingly, for the experimental values of $\tau_{\text{s}}^0 \approx 1.48\,\text{ns}$ ($B_{\text{ext}} = 0$) and $\tau_{\text{s}}^B \approx 0.52\,\text{ns}$ ($B_{\text{ext}} > 50\,\text{mT}$), respectively, the relative strengths of the varying SOC coefficients are determined via

$$\frac{\alpha_{\parallel}^2}{2\beta^2 + \alpha_{\perp}^2} \approx 4 \, , \tag{10}$$

which implies that the interface-induced z-contribution to the effective SO field $\mathbf{\Omega_k}$ is significantly larger than the x–y-contributions from both bulk ($\alpha_{\parallel} > \beta$) and the interfaces ($\alpha_{\parallel} > \alpha_{\perp}$).

Supplementary References

[1] Dresselhaus, G. Spin–Orbit Coupling Effects in Zinc Blende Structures. *Phys. Rev.* **100**, 580–586 (1955).

[2] Rashba, E. I. Properties of Semiconductors with an Extremum Loop. 1. Cyclotron and Combinational Resonance in a Magnetic Field Perpendicular to the Plane of the Loop. *Sov. Phys. Solid State* **2**, 1109–1122 (1960).

[3] Bychkov, Y. A. & Rashba, E. I. Properties of a 2D electron gas with lifted spectral degeneracy. *JETP Lett.* **39**, 78–81 (1984).

[4] Margulis, A. D. & Margulis, V. A. Spin relaxation of free carriers in semiconductors with the wurtzite structure. *Sov. Phys. Semicond.* **18**, 305–308 (1984).

[5] Lew Yan Voon, L. C., Willatzen, M., Cardona, M. & Christensen, N. E. Terms linear in k in the band structure of wurtzite-type semiconductors. *Phys. Rev. B* **53**, 10703–10714 (1996).

[6] Lo, I., Wang, W. T., Gau, M. H., Tsay, S. F. & Chiang, J. C. Wurtzite structure effects on spin splitting in GaN/AlN quantum wells. *Phys. Rev. B* **72**, 245329 (2005).

[7] Wang, W.-T. *et al.* Dresselhaus effect in bulk wurtzite materials. *Appl. Phys. Lett.* **91**, 082110 (2007).

[8] Fu, J. Y. & Wu, M. W. Spin–orbit coupling in bulk ZnO and GaN. *J. Appl. Phys.* **104**, 093712 (2008).

[9] Buß, J. H., Rudolph, J., Natali, F., Semond, F. & Hägele, D. Anisotropic electron spin relaxation in bulk GaN. *Appl. Phys. Lett.* **95**, 192107 (2009).

[10] Buß, J. H., Rudolph, J., Natali, F., Semond, F. & Hägele, D. Temperature dependence of electron spin relaxation in bulk GaN. *Phys. Rev. B* **81**, 155216 (2010).

[11] Rudolph, J., Buß, J. H. & Hägele, D. Electron spin dynamics in GaN. *Phys. Status Solidi B* **251**, 1850–1860 (2014).

[12] Cartoixà, X., Wang, L.-W., Ting, D.-Y. & Chang, Y.-C. Higher-order contributions to Rashba and Dresselhaus effects. *Phys. Rev. B* **73**, 205341 (2006).

[13] Tarasenko, S. A. Spin relaxation of conduction electrons in (110)-grown quantum wells: A microscopic theory. *Phys. Rev. B* **80**, 165317 (2009).

[14] Dyakonov, M. I. & Perel, V. I. Spin relaxation of conduction electrons in noncentrosymmetric semiconductors. *Sov. Phys. Solid State* **13**, 3023–3026 (1972).

[15] Meier, F. & Zakharchenya, B. P. (eds.) *Optical orientation*, vol. 8 of *Modern problems in condensed matter sciences* (North-Holland, Amsterdam, 1984).

[16] Žutić, I., Fabian, J. & Das Sarma, S. Spintronics: Fundamentals and applications. *Rev. Mod. Phys.* **76**, 323–410 (2004).

[17] Fabian, J., Matos-Abiague, A., Ertler, C., Stano, P. & Žutić, I. Semiconductor Spintronics. *Acta Phys. Slovaca* **57**, 565–907 (2007).

[18] Dyakonov, M. I. (ed.) *Spin Physics in Semiconductors*, vol. 157 of *Springer Series in Solid-State Sciences* (Springer, Berlin Heidelberg, 2008).
